# Deep learning-driven MRI for accurate brain volumetry in murine models of neurodegenerative diseases

**DOI:** 10.3389/fnins.2025.1632169

**Published:** 2025-11-03

**Authors:** Arno Doelemeyer, Saurabh Vaishampayan, Stefan Zurbruegg, Frédéric Morvan, Giuseppe Locatelli, Derya R. Shimshek, Nicolau Beckmann

**Affiliations:** ^1^Novartis Biomedical Research, Basel, Switzerland; ^2^École Polytechnique Fédérale de Lausanne, School of Engineering, Lausanne, Switzerland

**Keywords:** amyotrophic lateral sclerosis (ALS), artificial intelligence, deep learning, magnetic resonance imaging (MRI), multiple sclerosis (MS), neurodegeneration, volumetry, 3R principles

## Abstract

Brain atrophy as assessed by magnetic resonance imaging (MRI) is a key measure of neurodegeneration and a predictor of disability progression in Alzheimer’s disease and multiple sclerosis (MS) patients. While MRI-based brain volumetry is valuable for analyzing neurodegeneration in murine models as well, achieving high spatial resolution at sufficient signal-to-noise ratio is challenging due to the small size of the mouse brain. *In vivo* MRI allows for longitudinal studies and repeated assessments, enhancing statistical power and enabling pharmacological evaluations. However, the need for anesthesia necessitates compromises in acquisition times and voxel sizes. In this work we present the application of a deep-learning-based segmentation approach to the reliable quantification of total brain and brain sub region volumes, such as the *hippocampus*, *caudate putamen*, and *cerebellum*, from T_2_-weighted images with a pixel volume of 78x78x250 μm^3^ acquired in 4.3 min at 7 Tesla using a conventional radiofrequency coil. The reproducibility of the fully automatic segmentation pipeline was validated in healthy C57BL/6 J mice and subsequently applied to models of amyotrophic lateral sclerosis, cuprizone-induced demyelination, and MS. Our approach offers a robust and efficient method for *in vivo* brain volumetry in preclinical mouse studies, facilitating the evaluation of neurodegenerative processes and therapeutic interventions. The dramatic reduction in acquisition time achieved with our AI-based approach significantly enhances animal welfare (3R). This advancement allows brain volumetry to be seamlessly integrated into additional analyses, providing comprehensive insights without substantially increasing study duration.

## 1 Introduction

Brain atrophy, assessed through magnetic resonance imaging (MRI), is a crucial *in vivo* measure of neurodegeneration and a predictor of disability progression in conditions such as Alzheimer’s disease (AD) (Boublay et al., 2020; Traini et al., 2020; Zhu et al., 2022) and multiple sclerosis (MS) (Fujimori et al., 2020; Koch et al., 2022; Rocca et al., 2017). MRI-based brain volumetry has been widely used in clinical trials to evaluate the efficacy of disease-modifying therapies at a group level (De Stefano et al., 2017; Giorgio and De Stefano, 2013; Kappos et al., 2016; Kolind et al., 2023; Swanson et al., 2021).

Quantifying the volume of the entire brain or specific brain regions using MRI is also valuable for analyzing neurodegeneration in murine disease models. Given the small size of the mouse brain, achieving sufficient spatial resolution, contrast, and signal-to-noise ratio (SNR) for reliable segmentation of brain subregions is challenging and often requires long acquisition times, which can be prohibitive from an animal welfare perspective. An alternative approach involves performing volumetric analysis *ex vivo*, which benefits from greater spatial resolution and sensitivity due to the lack of constraints on imaging time, the use of radiofrequency (RF) coils tightly fitting the organ, and the absence of movement artifacts (Lerch et al., 2012). High SNR three-dimensional images of isolated brains with isotropic voxel volumes of approximately (25)^3^ µm^3^ have been obtained in several hours (Crater et al., 2022; Holmes et al., 2017; Ma et al., 2019), providing data suitable for sophisticated analyses. However, *ex vivo* tissue preparation, such as formalin fixation, is necessary to preserve the tissue during long scanning times, which may lead to distortions and shrinkage due to the removal of water and other fluids (Ma et al., 2019).

*In vivo* imaging avoids these drawbacks and allows for longitudinal analyses of structural changes. Repeated non-invasive assessments enhance the statistical power of experiments and enable pharmacological studies to evaluate therapy effects at multiple time points in the same mouse (Obrecht et al., 2023). However, in-life examinations require compromises, as animals must be anesthetized during acquisitions. Published mouse brain volumetry studies using 7 T or 9.4 T scanners with conventional RF coils report measurement times between 12 and 90 min per animal for voxel sizes ranging from (230)^3^ to (100)^3^ μm^3^ (Borg and Chereul, 2008; Holmes et al., 2017; Hussain et al., 2017; MacKenzie-Graham et al., 2012; Smith et al., 2018; Yoo et al., 2023). With helium-cooled cryoprobes that improve SNR (Baltes et al., 2009), acquisition times of 38 min and 6 min have been reported at 9.4 T for voxel volumes of 38x38x250 μm^3^ (Hamilton et al., 2019) and 68x68x300 μm^3^ (Pallast et al., 2019), respectively.

To perform volumetric analyses, semantic segmentation algorithms are employed to classify voxels in the acquired images according to anatomical regions. Registration based segmentation algorithms map the acquired images to a space of reference brain volumes (ATLAS) for which manual annotations are available (De Feo and Giove, 2019). Deep learning-based methods involve training neural networks to classify individual voxels. In animal brain models, these methods have been used for semantic segmentation to detect lesions (Valverde et al., 2020), perform skull-stripping (Porter et al., 2024), and conduct volumetric analyses of anatomical regions in mouse brains (De Feo et al., 2021). Image registration methods have also been utilized alongside deep learning models to generate reference datasets (Kohler et al., 2024). Additionally, deep learning based image reconstruction methods have decreased the examination time in clinical MRI, by reducing the required number of averages in the scans (Cochran et al., 2025; Ursprung et al., 2023) or by reconstructing from undersampled k-space data (Calivá et al., 2020; Sriram et al., 2020). Their success in the clinical arena points to a promising application to mouse brain imaging, where anesthesia requirements may demand compromises in acquisition time and voxel size. While previous publications have analyzed the performance of deep learning approaches on standalone test datasets, to the best of our knowledge, the adoption of these techniques for tracking longitudinal changes in animal models of brain pathology has received little attention in the literature. This is a crucial step toward evaluating their potential for application in the preclinical context.

In this work, we demonstrate how deep learning can be used to reliably quantify the volumes of brain regions such as the *hippocampus*, *caudate putamen*, and *cerebellum* in mice from T_2_-weighted images with a pixel volume of 78x78x250 μm^3^ acquired in 4.3 min at 7 Tesla using a conventional radiofrequency (RF) coil. Following validation in healthy C57BL/6 J animals, we evaluate its performance to quantify brain volumetric changes in longitudinal studies corresponding to models of cuprizone-induced demyelination in C57BL/6 J mice, transactive response DNA binding protein 43 (TDP43) mice modeling amyotrophic lateral sclerosis, and in C57BL/6 J mice within an EAE model.

## 2 Materials and methods

### 2.1 Statement on animal welfare

*In vivo* experimental procedures adhered to Swiss animal welfare regulations. The protocols and experiments were approved by the Cantonal Veterinary Office of Basel, Switzerland, under license numbers BS-2119 and BS-2711. Prior to approval by the Cantonal Veterinary Office the experimental protocols were submitted to an ethical committee by the authorities. The ethical committee is named officially “Kantonale Tierversuchskommission”. The authors complied with the ARRIVE 2.0 guidelines for animal experimentation (Percie du Sert et al., 2020), ensuring all assessments were conducted blind.

### 2.2 Animals

Female (*n* = 108) or male (*n* = 12) C57BL/6 J mice were obtained from Charles River Laboratories (Sulzfeld, Germany) or Envigo (Itingen, Switzerland). C57BL/6 J animals were incorporated into the following studies: *n* = 26 female mice were used for optimization, testing and reproducibility assessment of the neural network; *n* = 12 female and *n* = 12 male mice served as wildtype controls for the TDP43 transgenic mice; *n* = 14 (*n* = 4 controls, *n* = 10 immunized) and *n* = 56 (*n* = 21 controls, *n* = 35 cuprizone) female mice were used in the EAE and the cuprizone model, respectively.

Prp-hTDP43*Q331K transgenic mice (*n* = 12 female, *n* = 12 male), expressing myc-tagged human TDP43 with the ALS-linked Q331K mutation, were bred in-house as described by Arnold et al. (2013). The founders underwent extensive genetic characterization (targeted locus amplification sequencing) to ensure genetic and phenotypic homogeneity. This revealed an insertion point in chromosome 4 without affecting the coding sequence. Founders with 6 transgenes were selected to establish our in-house colony, which may differ from that of Arnold et al. (2013) in the number of integrated copies. Immunohistochemistry against TDP43 was used to confirm both transgene expression and localization.

All mice were pathogen-free and housed in groups within individually ventilated cages, maintained on a 12/12-h light/dark cycle, with free access to standard chow.

### 2.3 Animal models

#### 2.3.1 TDP43 transgenic mice

Male/female control and Prp-hTDP43*Q331K mice were 8 weeks old at the start of the experiments. Neurofilament-light (NF-L), a marker of axonal damage (Khalil et al., 2018), was assessed in blood plasma (see description below). Histological detection of TDP43 in the brains of Prp-hTDP43*Q331K animals confirmed the pathology. Following an MRI acquisition animals were euthanized by a high dose of isoflurane. Brains designated for histology were removed from the skull and fixed in 4% paraformaldehyde for 48 h at 4 °C. TDP43 protein was detected through automated immunohistochemistry performed on the Ultra Discovery XT platform (Ventana, Roche Diagnostics, Rotkreuz, Switzerland). This process involved deparaffinization, rehydration, and heat-induced epitope retrieval in 10 mM citrate buffer at pH 6 and 95 °C for 8 min. The primary antibody for TDP43 detection (reference DH0016, Abnova, Taipei, Taiwan) was used at a 1:32,000 dilution and detected with the anti-mouse OmniMap detection kit (Roche Diagnostics). After chromogenic revelation with DAB, slides were counterstained with Hematoxylin. All reagents used in the platform were sourced from the Roche catalogue.

#### 2.3.2 Cuprizone-induced demyelination

Female C57BL/6 J mice were treated with cuprizone for 5 weeks as described elsewhere (Beckmann et al., 2018, 2023). Cuprizone (Bis(cyclohexanone) oxaldihydrazone, Sigma-Aldrich, Buchs, Switzerland) was incorporated into rodent food pellets (0.2% w/w) by Provimi Kliba AG (Kaiseraugst, Switzerland). Animals were 3, 6 or 16 months old at the beginning of the study. The effect of cuprizone was verified by quantifying the magnetization transfer ratio (MTR) in the *corpus callosum,* as detailed in Beckmann et al. (2018, 2023).

#### 2.3.3 Experimental autoimmune encephalomyelitis (EAE)

Eight-week-old female C57BL/6 J mice were immunized following the protocols described by Oliver et al. (2003) and Smith et al. (2018). Briefly, mice received a subcutaneous injection of recombinant myelin oligodendrocyte glycoprotein (in-house produced MOG1–125; 200 μg/100 μL) emulsified in 4 mg/mL complete Freund adjuvant (Sigma-Aldrich, Buchs, Switzerland) on the lower back. *Pertussis* toxin (Fluka; 100 ng per mouse) was administered intraperitoneally on days 0 and 2. Disease status was monitored daily using a scoring system: 0 - normal appearance; 1 - complete tail paralysis; 2 - unilateral partial hind limb paralysis; 3 - complete bilateral hind limb paralysis; 4 - quadriplegia; 5 - death. Scoring was performed by a trained individual blinded to the treatment groups.

Additionally, repeated blood sampling from the lateral saphenous vein was conducted for longitudinal neurofilament-light (NF-L) assessment. Whole blood was drawn using EDTA-coated capillary tubes (CB300, Sarstedt, Nümbrecht, Germany), placed on ice, and centrifuged at 2000 g for 10 min at 4 °C. Plasma was carefully removed using a pipette, separated into aliquots, flash frozen in liquid nitrogen, and stored at −80 °C until use. NF-L levels in mouse plasma were determined using a commercially available NF-Light kit (Quanterix, Lexington, MA, catalog # 103186) on the SIMOA HD-1 analyzer (Quanterix). Plasma samples were diluted 1:40 with NF-Light sample diluent provided in the kit, loaded into the HD-1 analyzer, and assessed in duplicate according to the manufacturer’s instructions.

### 2.4 MRI acquisitions

Measurements were conducted using a Biospec 70/30 spectrometer (Bruker Medical Systems, Ettlingen, Germany) operating at 7 Tesla. Images were acquired from anesthetized, spontaneously breathing mice using a brain circularly polarized coil (Bruker, Model 1P T20063 V3; internal diameter 23 mm) for radiofrequency (RF) excitation and detection. Neither cardiac nor respiratory gating was applied. After a brief introduction with 3–5% isoflurane (Piramal Pharma, Mumbai, India) in a box, animals were maintained under anesthesia with 1.5% isoflurane in oxygen, administered via a nose cone. During acquisitions, animals were positioned prone in a Plexiglas cradle, with body temperature maintained at 37 ± 1 °C using a heating pad, and respiration monitored.

Images for machine learning network training were obtained using a T_2_-weighted, two-dimensional (2D) multislice RARE (Rapid Acquisition with Relaxation Enhancement) sequence (Hennig et al., 1986) with the following parameters: effective echo time (TE) 49.9 ms, minimum TE 12.48 ms, echo spacing 12.48 ms, repetition time (TR) 8 s, RARE factor 8, field of view (FOV) 30 × 20 mm^2^, matrix size 384 × 256, pixel size 0.078 × 0.078 mm^2^ and slice thickness 0.25 mm. A total of 32 adjacent coronal slices covered the whole brain. Hermite pulses of duration/bandwidth 1 ms/5400 Hz and 0.64 ms/5344 Hz were used for RF excitation and refocusing, respectively. Magnetization transfer contrast was introduced by a gauss pulse of 1 ms/2740 Hz duration/bandwidth placed 3.5 ms before the excitation pulse and applied with a 3 μT RF peak amplitude, a 1,500 Hz irradiation offset, one pulse per TR. Acquisitions were performed with either one average (NEX = 1, 4 min 16 s acquisition time) or 6 averages (NEX = 6, 25 min 36 s acquisition time).

A 2D multislice gradient-recalled FLASH (Fast Low-Angle Shot) acquisition (Haase et al., 1986) was used to assess the magnetization transfer ratio (MTR), a measure reflecting myelin content (Beckmann et al., 2018, 2023; Giorgetti et al., 2019). The parameters of the FLASH sequence were: TE/TR 2.8/252.8 ms, FOV 20 × 18 mm^2^, matrix size 213 × 192, pixel size 0.094 × 0.094 mm^2^, slice thickness 0.5 mm, 15 adjacent slices, 4 averages. A hermite pulse of 0.9 ms/6000 Hz duration/bandwidth and flipangle 30° was used for radiofrequency excitation. MTR contrast was introduced by a gauss pulse of 15 ms/182.7 Hz duration/bandwidth applied with RF peak amplitude of 7.5 μT and an irradiation offset of 2,500 Hz. The acquisition was then repeated with the same parameters but without the introduction of the MTR contrast. MTR was computed using the formula MTR = (S_0_ − S_MTR_)/S_0_, where S_0_ and S_MTR_ represent, respectively, the signal intensities in the FLASH acquisitions without and with the introduction of the MTR contrast. The total acquisition time for both data sets was 6 min 31.6 s.

### 2.5 Semantic segmentation of MRI data: training a convolutional neural network

**Figure 1:**
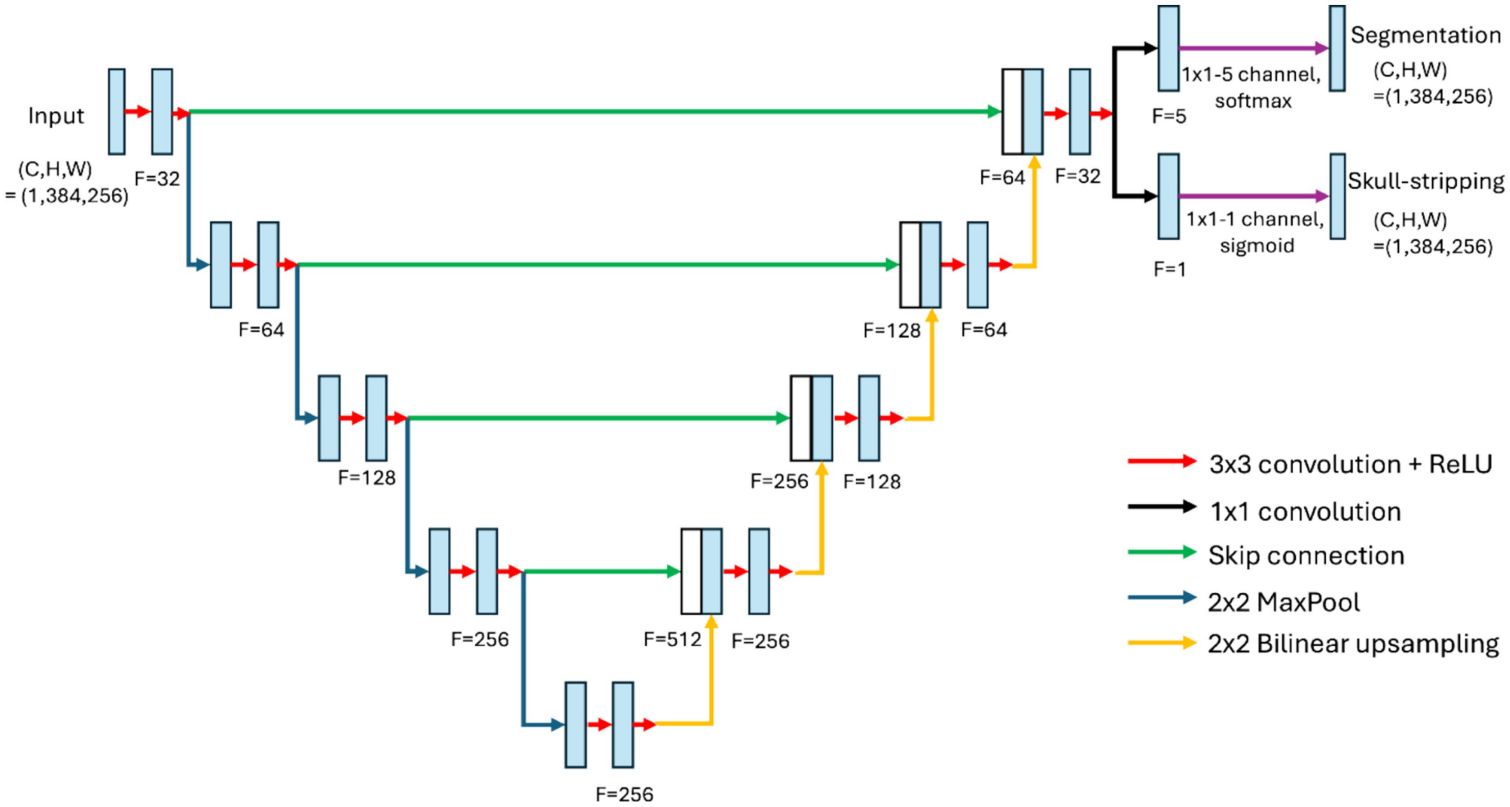
Architecture of the modified U-Net used in present work.

In this work, we utilized a modification of the U-Net architecture (Ronneberger et al., 2015) for configuring the neural network. The network performs two tasks: skull-stripping and semantic segmentation. The input to the network consisted of 2D coronal images (384 × 256 pixels) acquired using the previously described RARE protocol. The network consists of an encoding part, which downscales an image to a 24 × 16 × 256 latent space, followed by a decoding part that upscales it to a 384 × 256 × 32 size. After this step, similarly to multiple U-Net (MU-Net) (De Feo et al., 2021), the decoder bifurcates into two parts for performing skull-stripping and semantic segmentation, with the corresponding outputs obtained by applying 2D convolutions followed by sigmoid and softmax activation functions, respectively. The total number of trainable parameters for the neural network were about 4.9 millions. A scheme of the network can be found in Figure 1.

The semantic segmentation task consisted of classifying each voxel into one of five categories: “*Cerebellum*,” “*Hippocampus*,” “Ventricles,” “*Caudate Putamen*,” and “Remaining Brain Tissue.” To enable accurate segmentation for both small and large objects, the DICE loss function was applied while training the neural network, with the Adam optimizer for The model was implemented using the PyTorch Lightning framework (Falcon and The PyTorch Lightning Team, 2019).

In order to obtain the dataset size for model training with optimized resources, we employed an iterative approach. The model was trained with a dataset with manual annotations, followed by validation step consisting of visual inspection (QC step) by two observers, who identified whether there were significant discrepancies between tissue morphology and segmentation outputs. Following this, new images were annotated and added to the training and validation datasets, and the iterative process continued until the observers deemed the classifier’s performance sufficient on an unseen test dataset.

The final dataset after the convergence of the iterative approach, consisted of 28 annotated volumes, which was split into 23 volumes (736 images) for training and 5 volumes (160 images) for training and validation, respectively. The test dataset, which was used for QC evaluation, consisted of 125 volumes. Manual annotations to generate the dataset were performed using either 3D Slicer (https://www.slicer.org, Kikinis et al., 2014) or QuPath (https://qupath.github.io, Bankhead et al., 2017). Networks were trained *de novo* when additional training data was included in the iterative workflow. Furthermore, in order to improve model robustness, data augmentation consisting of random intensity and spatial transformations was applied during the training phase, with parameters summarized in Table 1.

**Table 1:** Data augmentation parameters.

Augmentation	Method	Range
Intensity	Gamma correction	0.64–1.25
	Gaussian blur	Kernel size = 5, σ ∈ [0.1,0.8]
	Rescale (brightness)	0.9–1.25
	Sharpness	1.0–2.0
Spatial	Rotation	−10^o^ to 10^o^
	Translation	+ − 10% (x and y axes)
	Shear	−5^o^ to 5^o^
	Scaling	0.75–1.25

During the training phase, transformations applied sequentially with parameters sampled from a uniform distribution in the corresponding range.

We also evaluated the impact of image noise levels (i.e., number of averages) on segmentation outcomes. Two datasets were acquired in the same session with the animals in the same position: a single average acquisition (4.3 min, NEX = 1) and an averaged acquisition (25.6 min, NEX = 6). Two networks were trained separately on single- and six-average datasets, respectively, and their performances were compared in terms of DICE scores. The outcomes were used to evaluate whether deep learning can be used for accurate segmentation on single averaged images.

### 2.6 Statistics

Brain volumetric data determined by MRI, in conjunction with deep learning, as well as MTR assessments, were analyzed using ANOVA with random effects (Systat version 13; Systat Software Inc., San Jose, California, USA) to account for the longitudinal structure of the data. Unpaired Student’s t-tests were performed using OriginPro (version 2023b; OriginLab Corporation, Northampton, MA, USA). A *p*-value of < 0.05 was considered significant.

## 3 Results

Quantitative and qualitative results for the deep learning model applied to mouse brain volumetry analyses are now presented. We begin by discussing the training and validation processes of the deep learning model for semantic segmentation. Additionally, we provide quality control and reproducibility assessments to evaluate the performance of the trained neural network. Following this, the trained network is applied to volumetric analysis of three animal models: TDP43 transgenic mice, EAE mice, and the cuprizone model.

### 3.1 Development of a deep learning-based semantic segmentation algorithm

#### 3.1.1 Supervised learning for semantic segmentation

**Figure 2:**
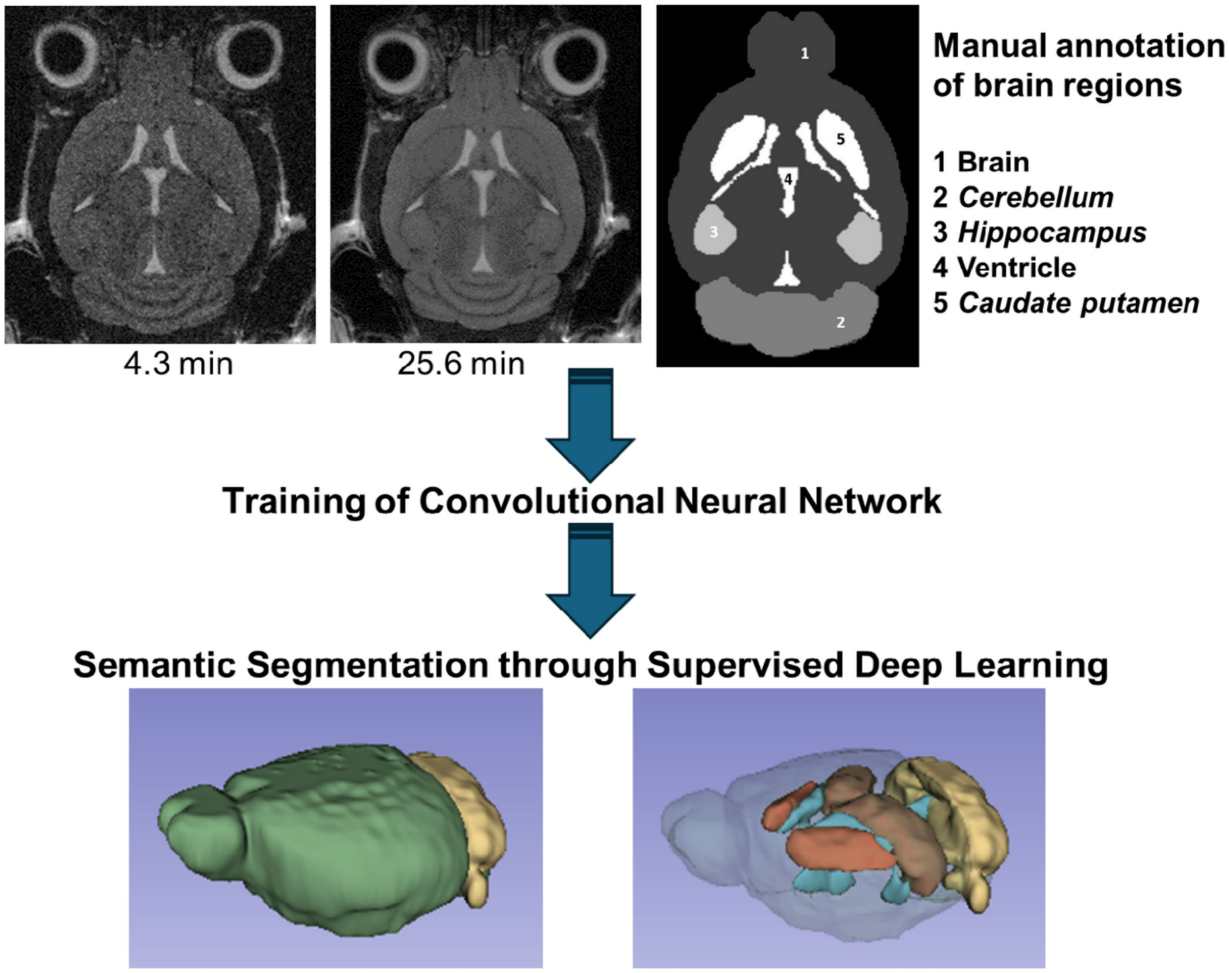
Workflow for semantic segmentation of the mouse brain using supervised learning. A convolutional neural network was trained on manually annotated T_2_-weighted images acquired in either 4.3 min or 25.6 min using a RARE sequence. The acquisition parameters were: effective TE 49.9 ms, TR 8 s, RARE factor 8, pixel size 0.078 × 0.078 mm^2^, slice thickness 0.25 mm, 32 adjacent coronal slices, 1 or 6 averages.

Figure 2 summarizes the workflow for the general deep learning-based segmentation of mouse brain MRI multislice datasets acquired using a conventional radiofrequency coil.

Two convolutional neuronal networks were trained on noisy (single average acquisition, 4.3 min) and noise-suppressed (NEX = 6 averaged acquisition, 25.6 min) datasets from control, healthy C57BL/6 J mice. Twelve datasets comprising 384 images were used for training and 192 images from 6 datasets were used for testing. Dice loss scores derived from test images showed a similar performance of both networks (0.977 for the 4.3-min and 0.976 for the 25.6-min acquisitions) for the whole brain volumetric assessments (Table 2). Based on this comparison we decided to use the single average 4.3 min acquisition protocol for all further investigations.

**Table 2:** Summary of test dataset DICE scores, comparing segmentation performance of neural networks trained on single acquisition (4.3 min, NEX = 1) and noise-suppressed (25.6 min, NEX = 6) T_2_-weighted MRI multislice images acquired from 6-month-old healthy C57BL/6 control mice (*n* = 160 images).

Region	DICE (NEX = 1)	DICE (NEX = 6)
Total brain	0.976	0.977
*Cerebellum*	0.912	0.924
*Hippocampus*	0.876	0.885
*Caudate putamen*	0.852	0.856
Ventricles	0.880	0.872

Values are in line with those published by De Feo et al. (2021).

**Figure 3:**
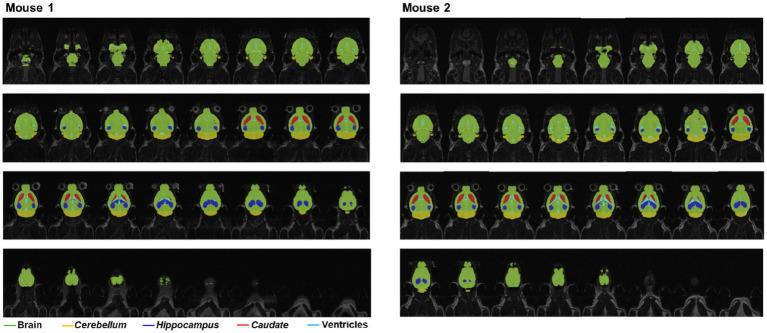
Deep learning-based semantic segmentation of mouse brain MRI images acquired in 4.3 min. Visualization of the segmented regions provided a basis for the quality control of the segmentation algorithm, as illustrated here by two representative examples.

Based on the single average trained network established above we utilized additional MRI data and the iterative workflow to create a new “production” classifier for volumetry, using a total of 896 images from 28 datasets for training the final optimized version of the network in two iteration steps. Application of this final classifier in a preliminary study using the MRI protocol outlined above showed a consistent performance in segmenting the total brain as well as sub-regions (Figure 3). An example of volumetric assessments is summarized in Table 3 for the healthy control study subgroup (7 mice). The obtained volumes are consistent with literature data (Jiang et al., 2024; Smith et al., 2018).

**Table 3:** Summary of brain volumetry based on deep learning analyses of T_2_-weighted MRI multislice images acquired in 4.3 min from 6-month-old healthy C57BL/6 control mice (means ± SD, *n* = 7 animals).

Region	Volume (μl)
Total brain	509.7 ± 8.1
*Cerebellum*	59.2 ± 2.0
*Hippocampus*	24.4 ± 1.4
*Caudate putamen*	18.8 ± 0.4
Ventricles	10.4 ± 1.5

Visual quality control of resulting segmentation, as illustrated in Figure 3, was conducted for every dataset acquired in 4.3 min that contributed to the results reported here. No relevant areas of segmentation errors were detected in any of the 13,056 processed images comprised in the studies described below, with the skull stripping part of the model performing particularly well showing no false positive areas in the skull and in general outside of the brain area The semi-transparent rendering of the sub-region classification allowed for a direct comparison to the region border image contrasts in the MRI slices. From these QC assessments, it was concluded that the parameters of the convolutional neural network had converged to an accurate solution, so that no additional training was deemed necessary.

#### 3.1.2 Repetitive assessments in naive C57BL/6 J mice

**Figure 4:**
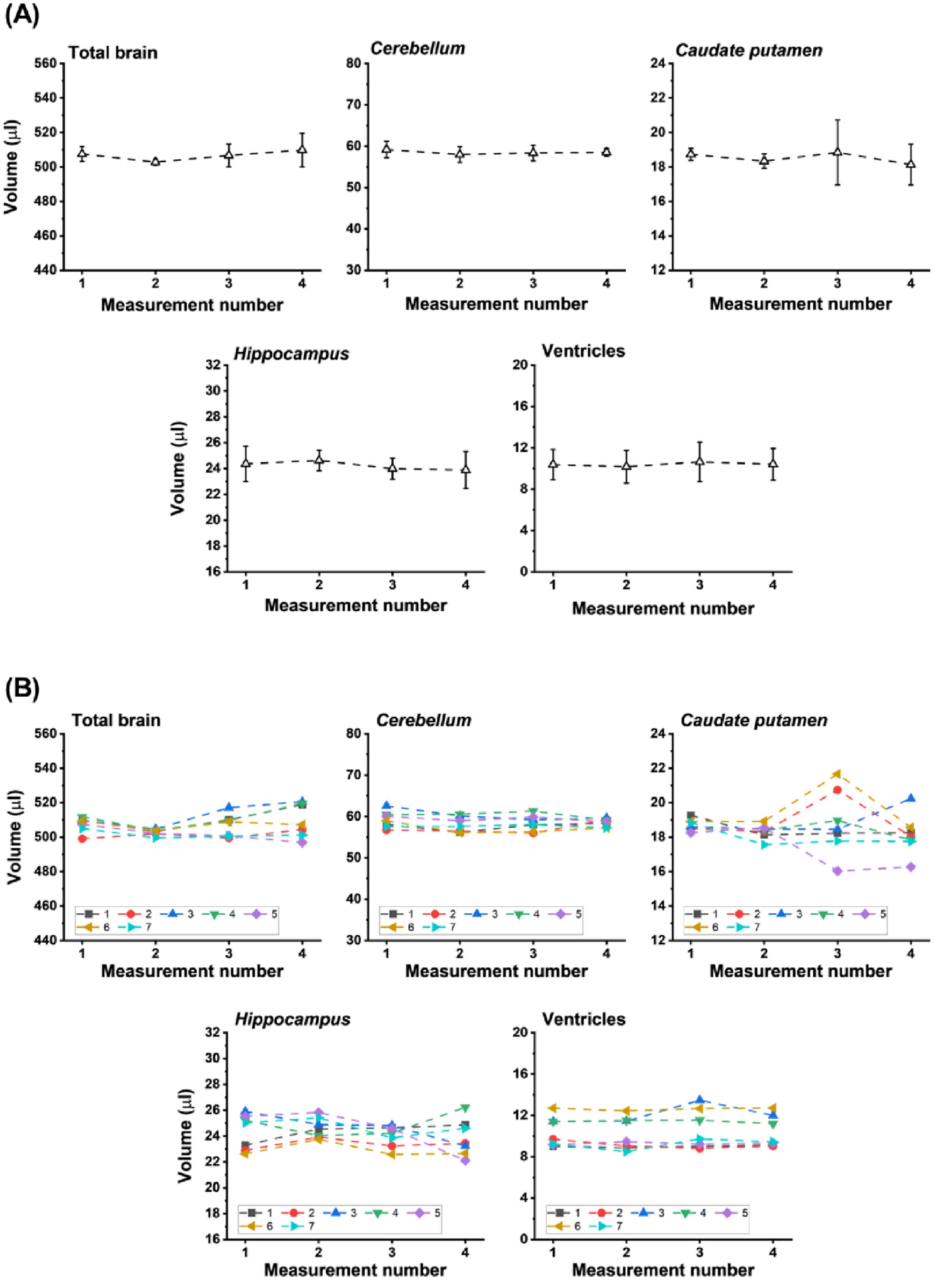
Reproducibility assessments in 6-month-old healthy C57BL/6 J mice measured once a week. **(A)** Volume (means ± SD, *n* = 7 animals) of total brain and of brain subregions determined from MRI data sets acquired in 4.3 min. **(B)** Individual values for each mouse at the different measurements.

Before applying the algorithm to brain volumetry in animal models, repeated assessments were conducted in 6-month-old naive C57BL/6 J mice, measured once a week for 4 weeks, to test the robustness of the measurements. Although an overall brain weight increase between 3 and 4 and 12 months of age has been reported for C57BL/6 male mice (Lessard-Beaudoin et al., 2015), the age of 6 months was considered reasonable for robustness tests, even if the first and last acquisitions were 1 month apart. Volumes derived using the deep learning approach resulted in stable measures for total brain and brain subregions (Figure 4). The mean coefficients of variation (COV) for the evaluations of various regions using images from the repeated acquisitions were: 0.9% (total brain), 1.8% (*cerebellum*), 3.6% (*hippocampus*), 3.3% (ventricles), and 5% (*caudate putamen*).

Given the low single-digit COV for repeated volumetric quantifications of total brain or brain subregions in healthy mice, further analyses were performed to investigate the sensitivity of the approach in detecting longitudinal changes in representative murine models of disease.

### 3.2 Applications of the deep learning-based volumetric assessment

#### 3.2.1 TDP43 transgenic mice

**Figure 5:**
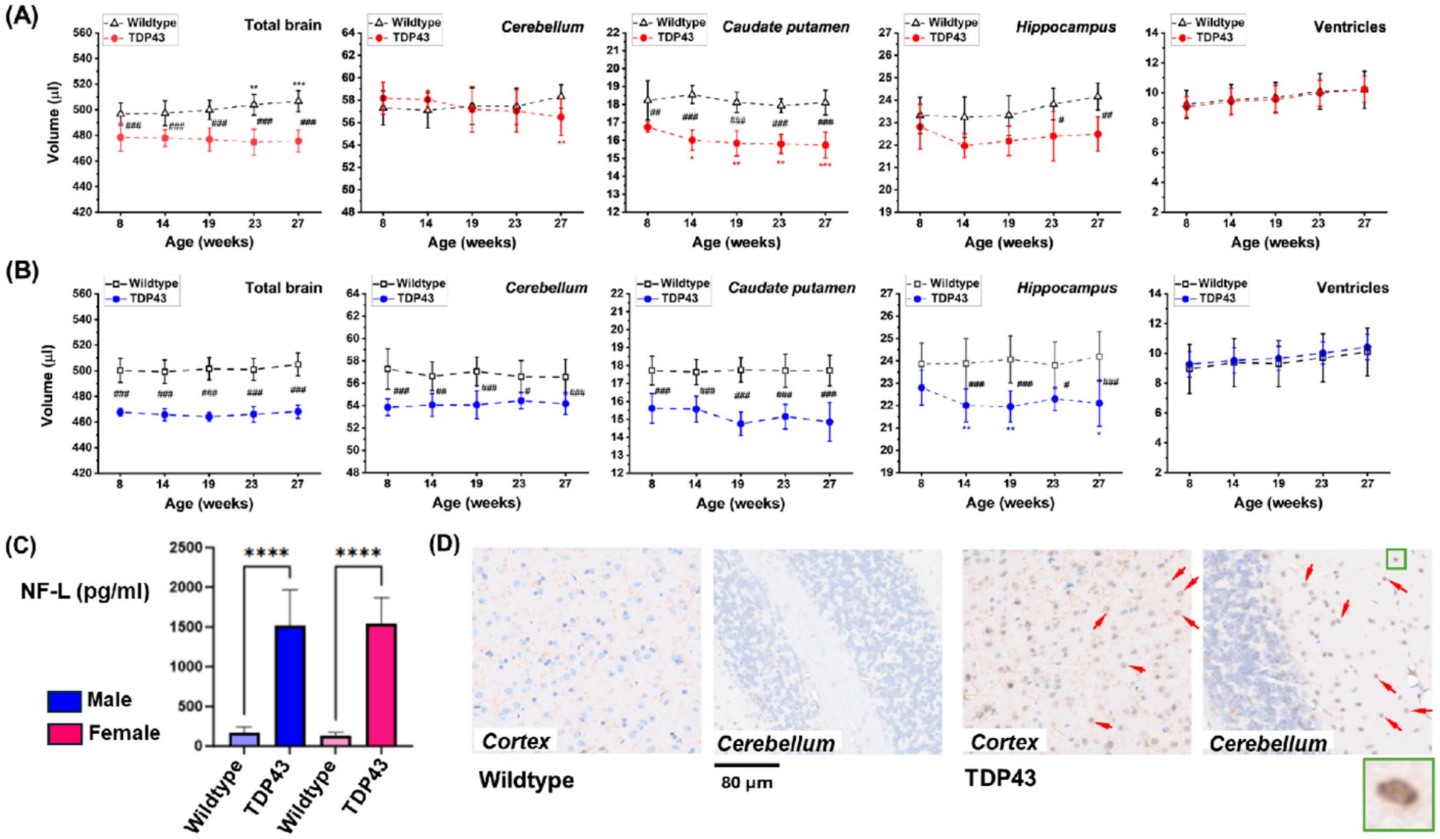
TDP43 transgenic mice. Longitudinal brain volumetry for **(A)** female and **(B)** male animals from MRI datasets acquired in 4.3 min. **(C)** Plasma NF-L at 4 months of age. Results are presented as means ± SD for *n* = 12 mice for each gender and genotype. Data were analyzed using ANOVA with random effects. Significance levels are indicated as follows: *p = 0.02, **0.001 < *p* < 0.01, ***p < 0.001 for comparisons to week 8 or week 10 values within the same group; #0.01 < *p* < 0.05, ##0.001 < *p* < 0.01, ###*p* < 0.001, and ****p < 0.0001 for comparisons between wildtype and TDP43 transgenic mice at different ages. **(D)** Representative histological sections of the cortex and cerebellum showing TDP43 staining in TDP43 transgenic animals only (arrows). The insert shows nuclear TDP43 staining.

Volumetric analyses in female animals revealed significantly smaller total brain, *caudate putamen*, and *hippocampus* volumes in TDP43 transgenic mice compared to age-matched wildtype female mice (Figure 5A). In male mice, the *cerebellum* of TDP43 transgenic mice also had a smaller volume compared to wildtype animals (Figure 5B). Increased plasma NF-L, a marker of axonal damage, was detected in both mutated males and females as early as 4 months of age (Figure 5C). Similar NF-L levels were present in older animals, indicating no clear progression. Histology at the end of the study revealed nuclear TDP43 staining in brain sections of TDP43 transgenic mice only (Figure 5D).

#### 3.2.2 EAE model

**Figure 6:**
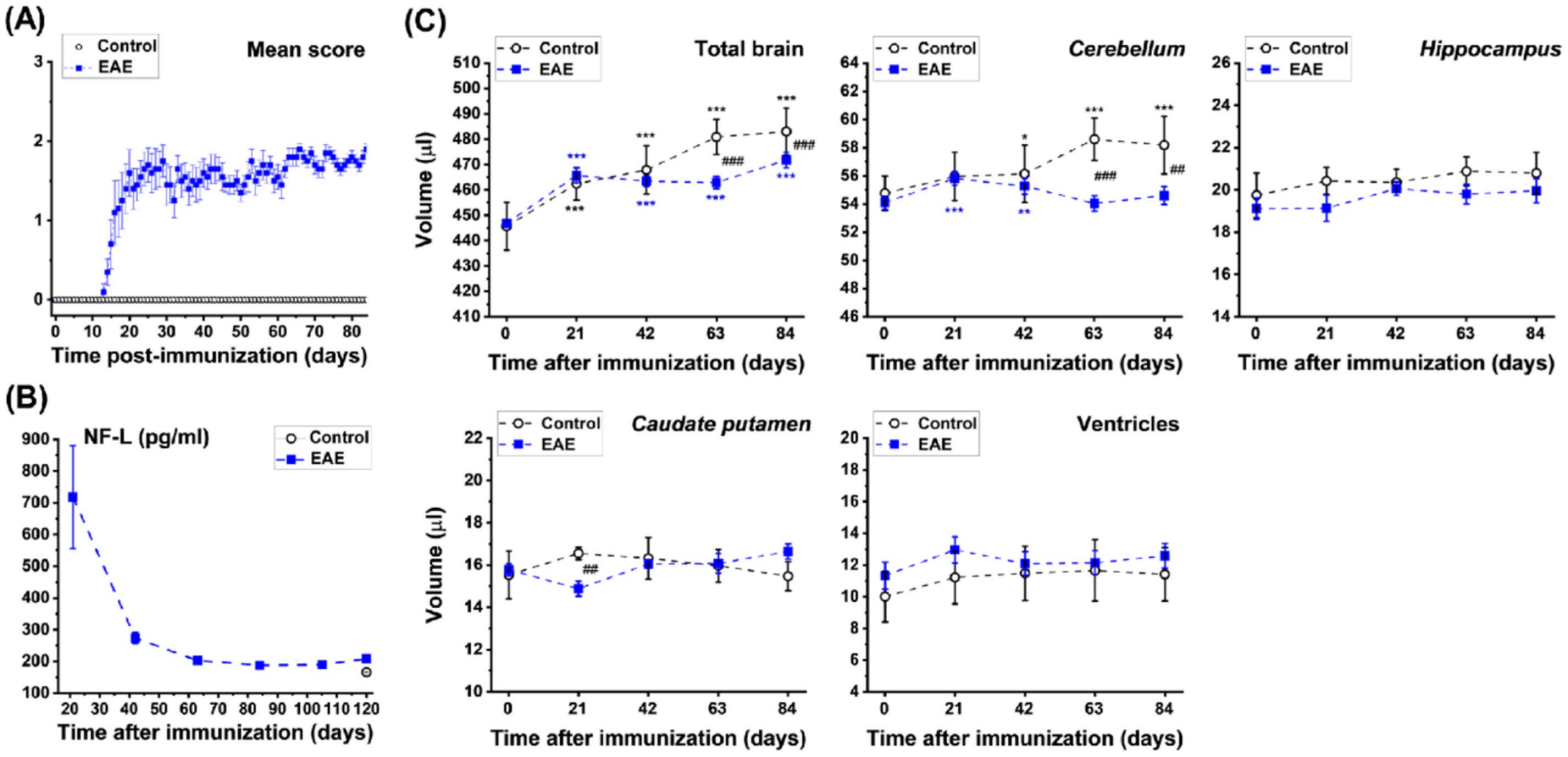
EAE model comprising female C57BL/6 J mice immunized with a subcutaneous injection of recombinant myelin oligodendrocyte glycoprotein (200 μg/100 μL). **(A)** Clinical scores and **(B)** plasma NF-L levels (means ± SEM, n = 4 controls, n = 10 immunized animals). **(C)** Volume (means ± SD) of total brain and brain subregions determined from MRI datasets acquired in 4.3 min from the same animals. Significance levels: *p = 0.038, **p = 0.005, ***p < 0.001 for ANOVA with random effects comparisons to baseline values within the same group; ##0.001 < *p* < 0.01 and ###*p* < 0.001 for comparisons between controls and EAE mice at the indicated ages.

Animals immunized with recombinant myelin oligodendrocyte glycoprotein developed chronic clinical pathology, as evidenced by increased motor impairment scores from day 14 post-immunization onwards (Figure 6A). Elevated levels of NF-L were detected in the blood plasma of EAE mice compared to naive, control mice throughout the experiment (Figure 6B). Deep learning-based analyses of images acquired from the same animals showed that, compared to age-matched controls, the total brain and *cerebellum* volumes of EAE-induced mice decreased starting at day 63 post-immunization (Figure 6C).

#### 3.2.3 Cuprizone model

**Figure 7:**
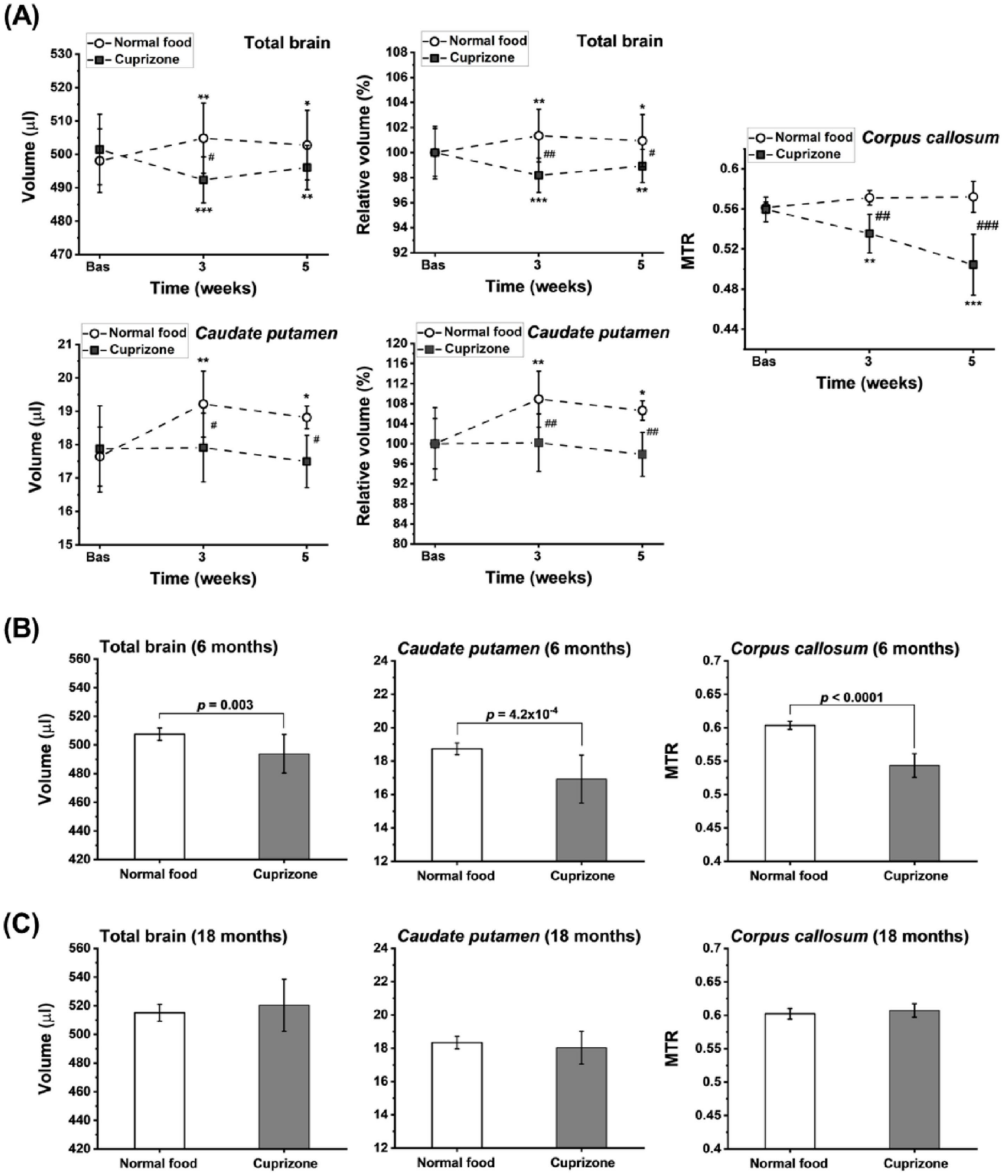
Age-dependent brain effects of cuprizone 0.2% delivered in food to female C57BL/6 J mice. **(A)** Volume and relative volume (means ± SD, *n* = 7 animals/group) of the total brain and caudate putamen determined from MRI datasets acquired in 4.3 min. On the right, MTR in the *corpus callosum* from the same animals, which were 3 months old at the beginning of the study. Significance levels: *0.01 < *p* < 0.05, **0.001 < *p* < 0.01, ***p < 0.001 for ANOVA with random effects comparisons to baseline values within the same group; #0.01 < *p* < 0.05, ##0.001 < *p* < 0.01, and ###*p* < 0.001 for comparisons between control food and cuprizone groups at the indicated time points. Same parameters (means ± SD) assessed after 5 weeks of normal food (*n* = 7 mice) or cuprizone feeding (*n* = 14 mice) for animals that were either 6 **(B)** or 18 months old **(C)** at the start of the experiment. Significance levels correspond to t-test comparisons.

Smaller total brain and *caudate putamen* volumes were observed in 3-month-old mice after 3 and 5 weeks of cuprizone intoxication (Figure 7A). Pathological changes were confirmed by significant reductions in the magnetization transfer ratio (MTR) in the *corpus callosum* of cuprizone-fed animals (Figure 7A), indicating demyelination induced by the copper chelator, as previously shown (Beckmann et al., 2018, 2023). Similar results were obtained in 6-month-old mice after 5 weeks of intoxication with the toxin (Figure 7B). However, neither brain volumetric changes nor MTR reduction in the *corpus callosum* were detected in 18-month-old animals following 5 weeks of cuprizone ingestion (Figure 7C). Age-dependent effects of cuprizone intoxication have been reported previously (Wang et al., 2013).

## 4 Discussion

MRI is inherently slower than other *in vivo* imaging modalities like ultrasound or computerized tomography due to the acquisition of multi-dimensional k-space data through one-dimensional signals. Over the past decades, efforts to accelerate MRI data acquisition have led to the development of compressed sensing, which enables accurate reconstruction from sparsely sampled k-space data (Feng et al., 2017; Ye, 2019). The FDA’s approval of compressed sensing protocols for clinical imaging attests to the maturity of this approach. Although applications to animals have emerged (Braig et al., 2020; Farias et al., 2018; Wang et al., 2020), routine use in small rodents remains challenging due to the technical optimization required for imaging small structures.

In this study, we pursued an alternative strategy to reduce the acquisition time for mouse brain images at 7 Tesla by using a standard MRI sequence and emphasizing deep-learning-assisted data evaluation. Compared to our previous work (Smith et al., 2018), we optimized the acquisition protocol by reducing the in-plane pixel size to 0.078 × 0.078 mm^2^. Further optimization of the RARE sequence, and/or the use of Fast Imaging with Steady-State Free Precession (FISP) as demonstrated by Gao et al. (2014), combined with deep learning-based image analysis, could lead to additional reductions in acquisition time. Despite being developed independently, our procedure resembles efforts recently reported by Man et al. (2023), demonstrating that deep learning enabled fast three-dimensional MRI of the human brain at low fields. Before applying the algorithm to brain volumetry in animal models, reproducibility assessments were performed in healthy mice. Based on these evaluations, we concluded that the approach demonstrated potential to detect minor volumetric changes expected under pathological conditions.

Following validation and reproducibility assessments, the deep learning-based segmentation approach was used for volumetric analysis of different animal models. The significantly reduced brain volume in TDP43 transgenic mice compared to age-matched wildtype mice (Figure 6) is consistent with the accumulation of TDP43 aggregates in the central nervous system, a common feature of many neurodegenerative diseases such as amyotrophic lateral sclerosis (ALS), frontotemporal dementia (FTD), and Alzheimer’s disease (AD) (Jo et al., 2020; Shen et al., 2023). Our results align with reports of atrophy and inflammatory events (astroglial and microglial reactivities) in both cortical (medial prefrontal *cortex*) and subcortical (*hippocampus*) structures in TDP43-related FTD mouse models (Santos-García et al., 2023; Tsai et al., 2010). Interestingly, brain atrophy was present early in our animal model, at 8 weeks of age. Recent studies have shown that transient protein folding targets aggregation in the early phase of TDP43-mediated neurodegeneration (San Gil et al., 2024).

Similar to multiple sclerosis (MS), the EAE model is characterized by inflammation and neurodegeneration in both the spinal cord and brain. Previous work revealed inflammation, demyelination, and neurodegeneration in several brain regions of EAE animals, including the *cortex* (Clark et al., 2016; MacKenzie-Graham et al., 2012), *caudate putamen* (Gentile et al., 2015), *cerebellum* (MacKenzie-Graham et al., 2009), *hippocampus* (Ziehn et al., 2010), and *corpus callosum* (Mangiardi et al., 2011). The significantly reduced total brain and *cerebellum* volumes in EAE mice compared to control animals (Figure 6) are consistent with earlier MRI volumetric assessments (Hamilton et al., 2019; MacKenzie-Graham et al., 2009, 2012; Smith et al., 2018). The significant increase in total brain volume during the study in both experimental groups align with reports of brain weight increases in C57BL/6 male mice up to 12 months of age (Lessard-Beaudoin et al., 2015).

In MS patients MRI has detected atrophy in the same brain areas as those mentioned before for the EAE model (see Matthews et al., 2023 for a recent review). MRI measures of atrophy have been proposed as a complementary approach to lesion assessment, facilitating the prediction of clinical outcomes and assessing treatment responses in MS (Sastre-Garriga et al., 2020). The question arises whether clinical learnings can be reproduced in the EAE animal model. Indeed, MRI volumetry has shown that a sphingosine-1-phosphate (S1P) receptor modulator had neuroprotective effects in MS patients (Kappos et al., 2015; Zivadinov et al., 2018) and in murine EAE (Smith et al., 2018), strengthening the translational/back-translational character of the approach. Performing MRI volumetry in the EAE model is thus crucial for preclinical testing of therapies aimed at reducing neurodegeneration. Deep learning has the potential to impact such studies by significantly reducing acquisition time, as illustrated in this work. This will be even more evident when adopting preclinical chronic models where animals are followed for longer periods to effectively screen novel compounds targeting the progressive phase of MS.

Copper dyshomeostasis has been linked to neurodegenerative diseases (Benetti et al., 2010; Gromadzka et al., 2020). This may explain the brain neurodegeneration observed in young (3-month-old) and adult (6-month-old) female mice receiving cuprizone for 5 weeks (Figures 7A,B), accompanied by significant reductions in MTR in the *corpus callosum*, indicative of demyelination induced by the copper chelator (Beckmann et al., 2018). Reduced total brain volume assessed *post-mortem* by the fluid displacement technique and a caliper in formalin-fixed samples has also been reported for the 6-week rat cuprizone model (Abbasi et al., 2024). Design-based stereology on formalin-fixed brain revealed significant volume reduction in the *corpus callosum* and various subcortical areas, particularly the internal capsule and thalamus, in male mice subjected to cuprizone intoxication for 12 weeks, while brain volumes were not altered after 5 weeks of cuprizone (Hochstrasser et al., 2019). Differences in gender and volumetric assessment conditions (*ex vivo* vs. *in vivo*) may contribute to discrepancies between the literature and our data. In particular, formalin fixation used to preserve brain tissue may lead to distortions and shrinkage (Ma et al., 2019). Further investigation is needed to understand the reduced *caudate putamen* volumes observed in young and adult cuprizone-challenged animals in two independent experiments (Figures 7A,B). The lack of response to cuprizone in 18-month-old mice (Figure 7C) is consistent with higher doses and prolonged feeding durations being required for robust demyelination in aged mice (Gingele et al., 2020; Wang et al., 2013). A higher resistance of mature oligodendrocytes in aged animals against cuprizone-induced apoptosis and reduced phagocytic capacity of aged microglia, resulting in delayed removal of myelin debris (Natrajan et al., 2015; Ruckh et al., 2012), may have contributed to the reduced effect of cuprizone in old mice.

While our work investigates the use of supervised deep learning models for mouse brain segmentation, other approaches have also been reported in the literature. For instance, Ma et al. (2014) developed a multi-atlas framework for rat brain segmentation. Recently, Kohler et al. (2024) demonstrated the potential of brain atlas-driven deep learning models on a large cohort of historical data, comprising more than 11,000 MRI datasets acquired from 9,660 adult rats over 10 years. In our case, we initially evaluated an atlas-based segmentation approach, based on the framework of Ma et al. (2014), to generate segmentation annotations for the deep learning model. However, this approach resulted in inadequate segmentation performance after training, with higher prediction errors near edges. Matching our data including the sub-areas of interest turned out to be challenging and especially for the ventricle and the *caudate putamen* we were not able to resolve substantial discrepancies and variations from animal to animal. Consequently, we resorted to manual annotation to generate the training dataset. Our iterative approach involved initial training, algorithm application, and the addition to the training set of manually curated annotations, where substantial differences between predictions and observed brain morphology occurred. This method allowed us to use a modest amount of training data while achieving good performance in detecting whole brain and subarea volumes. De Feo et al. (2021) trained an MU-Net convolutional neural network on manually segmented multislice RARE images, thereby achieving higher segmentation accuracy than state-of-the-art multi-atlas segmentation methods. Results were reported for mice of different ages and for various murine Huntington models. Compared to our work, MRI acquisitions performed at 11.7 T took 10.7 min per data set and the slice thickness of the images was of 0.6–0.7 mm. We evaluated the pre-trained MU-Net of De Feo et al. (2021) but it did not provide the expected segmentation results probably because of domain shifts, e.g., different slice thicknesses. There is broad consensus that voxel geometry plays a critical role in determining the volumetric properties of regions of interest. Since each voxel represents an average of the signals within its volume, increased slice thickness leads to the inclusion of signals from multiple tissue types, thereby introducing partial volume effects. This hampers the ability to distinguish between brain subregions and accurately delineate brain boundaries, ultimately reducing segmentation accuracy. While higher in-plane resolution (≤0.1 mm) in mouse brain MRI improves segmentation precision, particularly for small or thin anatomical structures, lower resolutions (≥0.15–0.2 mm) may be sufficient for larger regions (Ma et al., 2008). Notably, the use of more isotropic voxels, where in-plane resolution and slice thickness are more closely matched, has been shown to significantly enhance both segmentation accuracy and anatomical fidelity (Mulder et al., 2019). Therefore, the impact of more isotropic voxel configurations - such as thinner slices combined with lower in-plane resolution - achievable within a comparable scan time of 4 min, relative to the current voxel dimensions of 0.078 × 0.078 × 0.25 mm^3^, warrants further investigation.

It is important to note that in the present work the convolutional neural network used for segmentation was trained on images without apparent brain lesions. The selection of such datasets and the choice of brain areas for segmentation were dictated by our specific applications and were sufficient for the models of interest. However, for brains displaying tumors or edema, additional training would be required. Similarly, further training would be needed if additional brain regions need to be segmented and/or if there are changes in the geometric parameters of the images. While we anticipate that a moderate number of images would suffice for further training based on our experience with supervised learning, these are interesting directions for future work.

In summary, deep learning-based analyses enabled us to reduce MRI data acquisition time for volumetric assessment from 25 min to 4 min at 7 Tesla using a conventional radiofrequency coil and an established standard protocol, without compromising quality and robustness in detecting mouse brain tissue morphology. The low acquisition time, involving minimal exposure to anesthetics, represents a significant refinement of the experimental procedure, contributing to the 3Rs (refine, reduce, replace) concept of animal welfare (Beckmann and Ledermann, 2017; Obrecht et al., 2023; Russell and Burch, 1959). With this dramatic reduction in acquisition time, brain volumetry can now be easily added to further analyses in the same imaging session without significantly extending its duration. The fully automated image analysis approach is highly beneficial for pharmacological studies on neurodegeneration (Obrecht et al., 2023; Smith et al., 2018), allowing for easy randomization of animals into groups before treatment begins, based on MRI volumetry metrics.

## Data Availability

The datasets generated and/or analyzed during the current study are not publicly available due to internal regulations from Novartis on data availablity. Requests to access the datasets should be directed to arno.doelemeyer@novartis.com.
